# Localization and Biological Activities of Bioflavonoids from *Taxus canadensis* Marshall

**DOI:** 10.3390/ijms27125634

**Published:** 2026-06-22

**Authors:** Svetlana M. Zaytseva, Elena A. Kalasnikova, Rima N. Kirakosyan, Jing Liang, Elizaveta A Bolotina, Nikolay A. Trusov

**Affiliations:** 1 Department of Biotechnology, Russian State Agrarian University—Moscow Timiryazev Agricultural Academy, Timiryazevskaya Street, 49, Moscow 127434, Russia; r.kirakosyan@rgau-msha.ru (R.N.K.); dreawdas@163.com (J.L.); mouse-liza@mail.ru (E.A.B.); 2 Department of Cell Biology and Biotechnology, K.A. Timiryazev Institute of Plant Physiology, Russian Academy of Sciences, Moscow 127276, Russia; kalash0407@mail.ru; 3 Tsytsin Main Botanical Garden, Botanicheskaya Street, 4, Moscow 127276, Russia; n-trusov@mail.ru

**Keywords:** aril, localization, megastrobiles, microstrobiles, phenolic compounds, seeds, secondary metabolites, *Taxus*

## Abstract

Relict yew plants (*Taxus* L.) are not only ornamental plants with valuable wood but also have the ability to synthesize the unique compound taxol, which is successfully used in the treatment of cancer due to its powerful cytotoxic effect. Due to the presence of taxol, all parts of yew plants are extremely poisonous, but there have been cases where animals have eaten yew cones without fatal consequences. The biosynthesis of taxol is carried out due to the interaction of the isoprenoid and phenolic pathways of the secondary metabolism of plants. Despite the close attention of researchers to the peculiarities of taxol metabolism, there is very little data on the tissue and intracellular localization of both taxols and phenolic compounds in yew plants. Polyphenols are known to be physiologically active mediators involved in respiration, photosynthesis, plant growth and development, as well as in the process of in vitro dedifferentiation. Since *Taxus* is a relict species and has a limited and hard-to-reach range in nature, technologies that allow yew plants to be restored without removing plant material from the natural environment are of great practical importance: overcoming deep physiological dormancy of seeds, microclonal reproduction and initiation of plant growth. In vitro cultures are possible sources of biologically active and medicinal products. The aims and objectives of this study are to determine the characteristics of the formation and localization of phenolic compounds with high biological activity in various organs of plants of the genus *Taxus* and to determine the biological activity of ethanolic extracts from this plant. The objects of this study were the generative organs of *Taxus canadensis*, collected during the entire growing season (April–October) from plants growing in the Moscow region. The localization of various classes of polyphenols was determined by histochemical methods using light microscopy. Histochemical studies have shown the abundant presence of polyphenols in yew megastrobiles, microstrobiles, cones, seeds and aril. Ethanolic plant extracts were used to determine the biological activity. Flavans were dominant in the aril at various stages of vegetation, which was confirmed by our biochemical and histochemical studies. Extractive substances of *T. canadensis* show high antibacterial activity, especially in its shoot extracts. Ethanolic extracts from plant shoots showed greater biological activity than seed extracts. Aril extracts had the lowest cytotoxicity.

## 1. Introduction

Currently, more than ten thousand substances of plant origin with pharmacological activity are known that can cause multiple therapeutic and physiological effects, including fatal ones [1,2]. The poisonous active effects of relict plants of the genus *Taxus* L. have long been noted not only in humans but also in animals, manifested in damage to the mucous membrane of the gastrointestinal tract and weakening of the heart and respiratory organs. It has been established that the lethal oral dose of yew leaves ranges from 0.6 to 1.3 g/kg of body weight. Death, as a rule, occurs quickly from respiratory depression. Cases have been described where animals died within the first hour after eating yew [3,4]. However, there are known cases of animals eating yew seed cones without fatal consequences, while some bird species constantly include them in their diet [5,6]. The toxicity of plants and various organs within a species can vary greatly depending on the habitat, climatic factors, growing conditions, growing season, and stage of ontogenesis. This effect is associated with the peculiarities of the metabolism of a plant organism, when, in addition to the reactions of primary metabolism, plants are characterized by the formation of substances of secondary origin [7].

Researchers give special attention to the study of the secondary metabolism of plants of the genus *Taxus* due to their unique ability to biosynthesize taxol and its isoforms, which are substances that exhibit high biological activity in the treatment of cancer [8,9,10]. The pharmacokinetic features of taxol are manifested in the ability to block the anaphase of mitotic division by binding to the cytoskeleton of a cancer cell. Taxol is a derivative of a terpenoid nature. According to the literature, the formation of taxol is characteristic of all representatives of the genus *Taxus*. It has been established that taxol derivatives are diterpenoids containing a 3-(N-methylamino)-3-phenylpropanoyl structure, which is a derivative of a phenolic substance. The structure of several dozen compounds of this class has been established. Their biosynthesis, as well as the biosynthesis of phenolic compounds, occurs with the participation of phenylalanine, which once again indicates a close relationship between the biosynthesis of isoprenoids and phenolic compounds [11].

In the biosynthesis of baccatin III in plants of the genus Taxus, 3-amino-3-phenylpropanoyltransferase (BAPT) indeed plays a key role in the attachment of the side chain, which is formed from phenylalanine with the participation of phenylalanine aminomutase (PAM). As noted in the literature, upon attachment of the side chain, taxane-C13-O-phenylpropanoyl-CoA transferase catalyzes the initial esterification of C13-OH with the help of β-phenylalanoyl-CoA to form N-debenzoyl-2′-deoxytaxol. This process is part of a complex metabolic pathway leading to the formation of taxoids, including paclitaxel (taxol). Phenylalanine aminomutase (PAM) is an enzyme that catalyzes the interconversion between α- and β-isomers of phenylalanine. In the context of taxoid biosynthesis, PAM participates in the conversion of phenylalanine into intermediates that serve as precursors of the baccatin III side chain [12,13].

It should be noted that of all the varieties of gymnosperms, only representatives of the genus *Taxus* are characterized by the formation of taxol and its derivative baccatin III [14]. Thus, taxol biosynthesis is a specific and unique feature of plants of the genus *Taxus*, and at the late stage of taxol assembly, its accumulation positively correlates with the accumulation of polyphenols. However, almost every stage of biosynthesis is limited by the availability of diverse precursors, which vary in location. Also, the sites of the biosynthesis, transportation and storage of taxol have different compartments and are associated with the sites of accumulation of polyphenols [15,16]. In addition to taxoids, other representatives of secondary metabolism are formed in yew plants, many of which have very high biological activity [17,18]. These compounds include alkaloids, phenolic compounds, lignans, essential oils, and waxes.

In turn, polyphenols are the most common representatives of the secondary metabolism of plants, the formation of which has been widely noted in plant organisms. Phenolic compounds are active metabolites that participate in the processes of photosynthesis, respiration, energy transduction, allelopathy, as well as cell protection from pathogens and various stress factors [19]. The biosynthesis and accumulation of secondary compounds, including those of a phenolic nature, are characterized by plasticity and depend on the species of plants, organs, stage of ontogenesis, and vegetation period [20]. All plants synthesize compounds of a phenolic nature, and some compounds of a simple structure (phenylpropanoids) are almost universal. The ability of polyphenols to oxidize to form quinine derivatives also determines their widespread use in pharmacology as biologically active substances with hepatoprotective, neuroregulatory, capillary-strengthening, choleretic, antibacterial, antiviral and antitumor properties [21,22,23,24]. Recent research suggests that polyphenols have the potential to be effective components in cancer therapy. Initially, their anticarcinogenic effect was thought to be due to their ability to neutralize free radicals. The potential of polyphenols is being considered with an emphasis on their mechanisms of action as non-toxic antitumor compounds that do not adversely affect healthy tissues. However, more recent evidence suggests that they also directly regulate carcinogenesis signaling pathways and interact with proteins that control the cell cycle. This mechanism of action involves suppressing cell proliferation, reducing oxidative stress in tissues, and activating apoptosis. Polyphenols can methylate the promoters of genes that regulate the cell cycle, apoptosis, transcription factor synthesis, and fatty acid metabolism [25,26].

It is not surprising that bioflavonoids, as therapeutic antitumor compounds, have attracted a lot of attention from researchers in recent years. For example, flavonoids modulate signaling pathways during cancer initiation or promotion, but their effects are also significant in the processes of cancer progression, including invasiveness of surrounding areas and the formation of distant metastasis [27,28,29]. The anti-invasive effect is related to the inhibition of the IL-6-linked signaling pathway, which has an important role in the progression, invasion, and metastasis of breast cancer [30]. The scientific literature discusses nanopreparation-based strategies that significantly enhance the delivery efficiency and bioavailability of flavonoids in cancer therapy [31]. In addition, the bioavailability of flavonoids can be increased by their conjugation with metal ions or structural modification by radiation, and the anti-cancer properties of flavonoids affect all stages of carcinogenesis.

However, data on the formation and especially localization of phenolic compounds in plants of the genus *Taxus* are extremely scarce. Despite the fact that yew plants are characterized by a high ability to synthesize phenolic compounds, different representatives of the genus *Taxus* differ in their ability to form these substances [32,33,34]. Complex physiological and biochemical processes occurring not only in plants of the genus *Taxus* but also in their various organs remain insufficiently studied. Judging by the few literature data, the accumulation of taxol in yew seed is insignificant, and it may be absent in aril [17,35].

Because there is scarce literature on the formation of phenolic compounds in plants of the genus *Taxus*, our research aimed to study the formation and localization of these substances in various organs of this genus (using *T. canadensis* Marshall as an example). In addition, there is an opinion that the seeds of *Taxus* are edible, so it is important for us to compare the cytotoxic effect of the generative and vegetative organs of yew. The lack of available data on the formation of secondary metabolites in the generative organs of yew at various stages of vegetation also served as an incentive for our research. By learning the bacteriostatic and antifungal potential of plants of the genus *Taxus*, we will be able to come closer to understanding the reasons for the amazing longevity of these plants.

## 2. Results

### 2.1. Localization of Phenolic Compounds of Taxus

**Figure 1:**
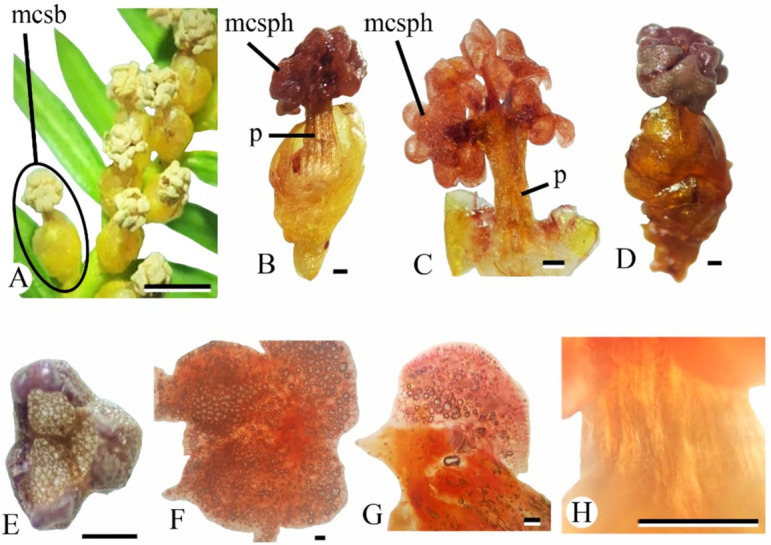
Microstrobiles and microsporophylls of Canadian yew. (**A**)—shoot with microstrobiles; (**B**,**C**)—localization of soluble phenolic compounds in microstrobile (reaction with Fast Blue reagent); (**D**,**E**)—localization of flavans in microstrobile; (**F**)—localization of flavans in microsporophylls (reaction with vanillin reagent); (**G**)—localization of soluble phenolic compounds in microsporophylls; (**H**)—microstrobile pedicel and localization of phenolic compounds in it (in the conductive tissues). **mcsb**—microstrobiles; **mcsph**—microsporophylls; **p**—microstrobile pedicel; Scale bar: (**A**)—1 cm, (**B**–**E**)—1 mm, (**F**–**H**)—0.01 mm.

The microstrobiles of the Canadian yew are spherical in shape on short legs and are covered with very small, filmy scales—scaly leaves. There are from 6 to 14 corymbose microsporophylls with a microsporangium on each strobe. Single megastrobiles are located at the ends of the short, strongly reduced axillary shoots (Figure 1A).

Specific histochemical reactions demonstrate the localization of polyphenols in microstrobiles, where they are singly located in the integumentary and conductive tissues of filmy scaly leaves. A fairly vivid reaction to the total content of soluble phenolic compounds is observed in microsporophylls, in cell walls, and in the form of amorphous and finely granular inclusions (Figure 1B,C,G). However, the reaction to flavans in microsporophylls is fragmentary (Figure 1D,E). This may indicate that the phenolic complex of microsporophyll includes various classes of phenylpropanoid and flavonoid polyphenols. The stem of the microstrobile in epidermal cells and conductive tissues contains polyphenols in the cell walls and in the contents of vacuoles in the form of an amorphous substance (Figure 1H). The most pronounced reaction to flavans is typical for conductive microstrobile tissues.

**Figure 2:**
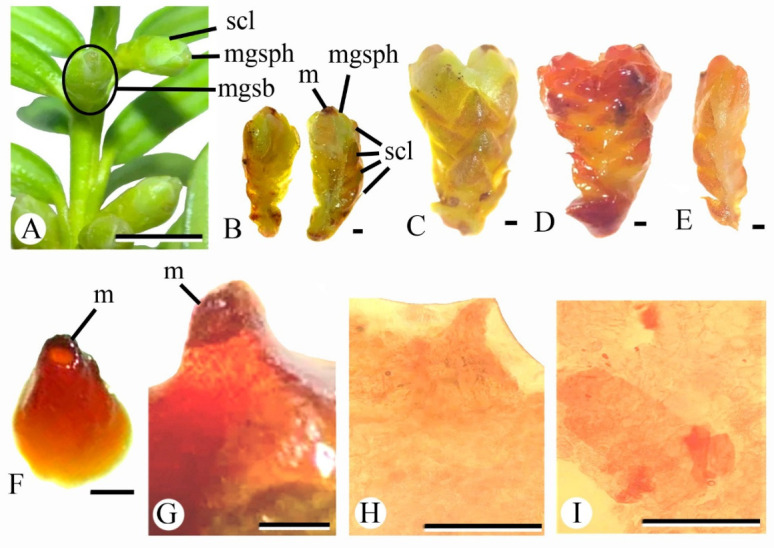
Megastrobiles and megasporophylls of Canadian yew. (**A**)—shoot with megastrobiles; (**B**,**C**)—localization of soluble phenolic compounds in megastrobile (in the scaly leaves of microstrobiles and in the micropyle); (**D**,**E**)—localization of flavans in megastrobili (vivid reaction in the scaly leaves); (**F**)—localization of soluble phenolic compounds in megasporophylls (in the area near the micropyle); (**G**)—localization of flavans in megasporophylls; (**H**,**I**)—localization of flavans in the ovule in the area near the micropyle (phenolic compounds are found in intercellular area and vacuoles idioblasts). **mcsb**—microstrobiles; **mcsph**—microsporophylls; **m**—micropyle; **mgsb**—megastrobili; **mgsbh**—megasporophylls; **scl**—scaly leaves of microstrobiles; Scale bar: (**A**)—1 cm, (**B**–**F**)—1 mm, (**G**–**I**)—0.01 mm.

In the scaly leaves of female cones (Figure 2A), the content of polyphenols, as well as in the scaly leaves of microstrobiles, is noted in the integumentary and conductive tissues. However, the reaction to flavans in the scaly leaves of megastrobiles is more pronounced than in microstrobiles (Figure 2D,E,G). The aril rudiments, integument, nucellus, and endosperm contain phenolic compounds, including flavans (Figure 2B,C,F). A brighter reaction is typical for micropiles. Flavans in the micropyle were predominantly present in the cell walls, but groups of cells with them were found in vacuoles in the form of amorphous matter and finely granular inclusions. At the same time, staining with Fast blue reagent for the total content of soluble polyphenols in micropyle was more intense and contrasting compared with histochemical reactions to flavans (Figure 2H,I).

Oviposition in yew occurs in late autumn, a year before fertilization, and overwinters during the formation of megasporocytes. The development of the male gametophyte in yew is long, about six months. After pollination (in March) and subsequent fertilization (late May–early June), the seeds ripen within two months but remain on the plant for a long time. The yew seed is surrounded by a fleshy, flanked outgrowth (aril), which is a modified megasporophyll that changes color from green to bright red as the seed matures. The epidermis and the multilayered parenchyma are isolated in the aril. According to literature data, it is the aril that determines the conditional edibility of mature yew seed cones for some bird species, which may be indirect evidence that the aril has the lowest content/absence of taxol compared to other plant organs [36,37]. Some researchers attribute the reported cases of harmless seed eating to the lack of digestive enzymes in birds that can overcome seed hardness. In addition, in medical practice, there have been cases of mammals successfully eating a small amount of yew seed cones, provided they get rid of the seed in advance.

**Figure 3:**
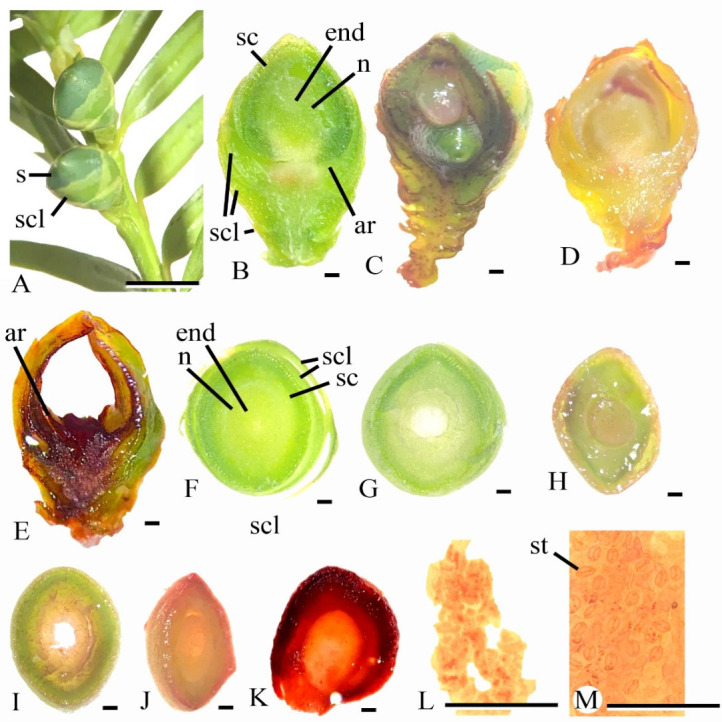
Immature seeds of Canadian yew and details of their structure. (**A**)—yew shoot with seeds at the early stages of development; (**B**)—longitudinal section of unstained immature seed; (**C**)—localization of polyphenols in immature seeds (reaction with Fast Blue reagent); (**D**)—localization of flavans in immature seeds (reaction with vanillin reagent); (**E**)—seed cone without seed, localization of total content of phenolic compounds in the primordium of aril (reaction with Fast Blue); (**F**,**G**)—cross-sections of unstained immature seed; (**H**,**I**)—cross-sections of immature seed with localization of soluble phenolic compounds (reaction with Fast Blue); (**J**,**K**)—cross-sections of immature seed with localization of flavans (reaction with vanillin reagent); (**L**)—localization of flavans in endosperm cells of developing seed; (**M**)—localization of flavans in the guard (closing) cells of stomata of scales. **scl**—scaly leaves of microstrobiles; **s**—seed; **sc**—seed coat; **end**—endosperm; **n**—nucellus; **ar**—arill; **st**—stomata; Scale bar: (**A**)—1 cm, (**B**–**K**)—1 mm, (**L**,**M**)—0.01 mm.

**Figure 4:**
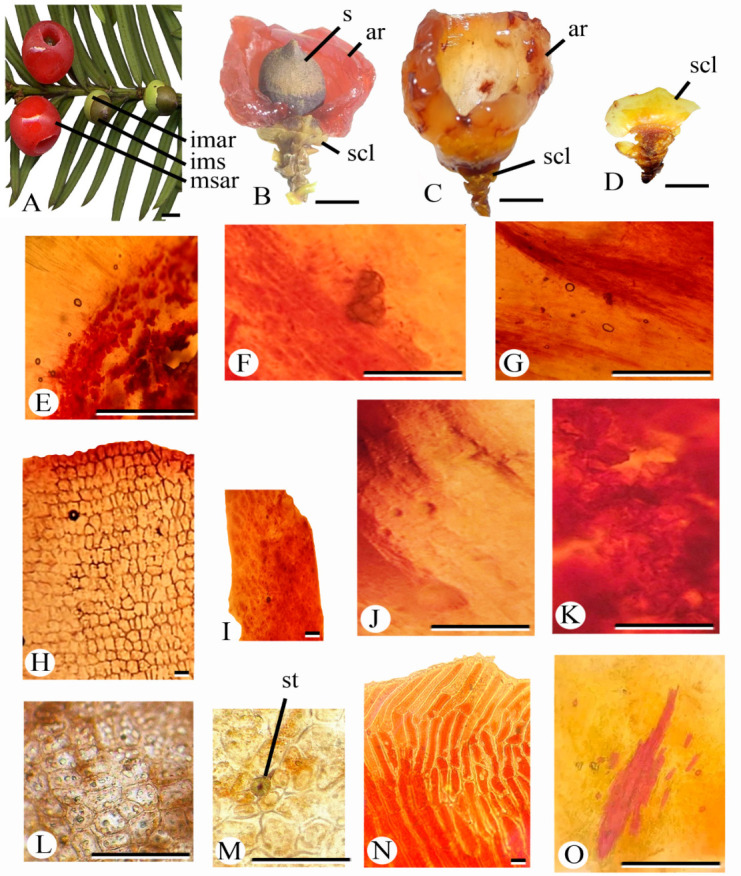
Mature seeds of Canadian yew and details of their structure: arils and scales. (**A**)—yew shoot with mature and immature berries; (**B**)—mature seed cone with partially removed aril; (**C**)—reaction to determine the localization of the total content of phenolic compounds in the aril (with Fast Blue reagent); (**D**)—reaction for polyphenols with Fast Blue reagent in scales; (**E**–**G**)—part of the aril adjacent to the chalaza, reaction to determine the localization of the total content of phenolic compounds (with Fast Blue reagent); (**H**,**I**)—the upper part of the aril (epidermis in plants), reaction to determine the localization of the total content of phenolic compounds (with Fast Blue reagent); (**J**,**K**)—part of the aril adjacent to the chalaza, reaction to determine the localization of flavans; (**L**,**M**)—reaction to determine the localization of the total content of phenolic compounds in the epidermis of the aril and in the guard (closing) cells of stomata; (**N**)—reaction to polyphenols with Fast Blue reagent in the epidermal cells of scales; (**O**)—reaction to flavans with vanillin dye in scales. **imar**—immature aril seeds; **ims**—immature seed; **msar**—mature aril seeds; **ar**—arill; **st**—stomata; **scl**—scaly leaves of microstrobiles; **s**—seed; Scale bar: (**A**–**D**)—1 cm, (**E**–**O**)—0.01 mm.

**Figure 5:**
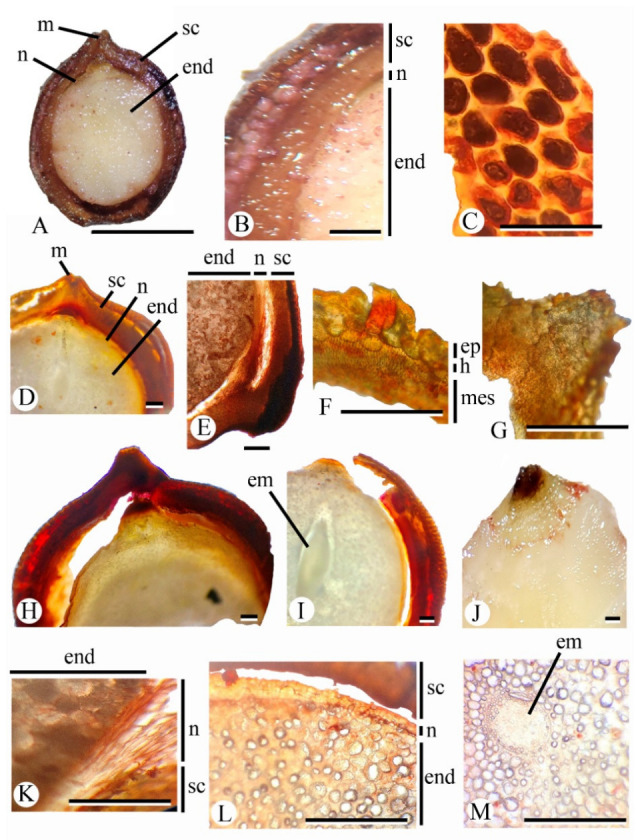
Mature seeds of Canadian yew and details of their structure: seed coat, remains of nucellus, endosperm and embryo. (**A**)—longitudinal section of seed; (**B**)—reaction for polyphenols with Fast Blue reagent in seed coat and endosperm (longitudinal section); (**C**)—epidermal cells of seed coat in plan, reaction for polyphenols with Fast Blue reagent (sclereids); (**D**)—seed fragment in the micropyle area, reaction for determination of localization of total phenolic compounds content (with Fast Blue reagent); (**E**)—seed fragment in the chalaza area, reaction for determination of localization of total phenolic compounds content; (**F**)—seed coat fragment in the middle part of seed, reaction for determination of localization of total phenolic compounds content; (**G**)—seed coat fragment in the micropyle area, reaction for determination of localization of total phenolic compounds content (with Fast Blue reagent); (**H**,**I**)—section of the seed in the micropyle area, a reaction to determine the localization of flavans; (**J**)—fragment of the endosperm and the remains of the nucellus in the micropyle area, a reaction to polyphenols with the Fast Blue reagent; (**K**)—remainder of the nucellus in the chalaza area, a reaction to determine the localization of the total content of phenolic compounds; (**L**)—fragment of the nucellus and endosperm, a reaction to determine the localization of the total content of phenolic compounds; (**M**)—fragment of the endosperm and embryo, a reaction to determine the localization of the total content of phenolic compounds (phenolic compounds are found in intercellular area). **m**—micropyle; **ep**—epiderma; **h**—hypoderma; **mes**—mesoderm; **n**—nucellus; **sc**—seed coat; **end**—endosperm; **em**—embryo; Scale bar: (**A**)—1 cm, (**B**,**D**,**E**,**H**–**J**)—1 mm, (**C**,**F**,**G**,**K**–**M**)—0.01 mm.

**Figure 6:**
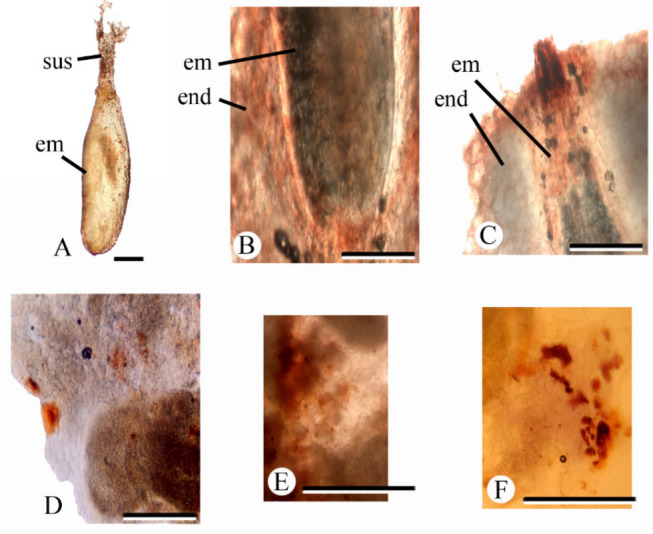
Mature seeds of Canadian yew and details of their structure: embryo. (**A**)—embryo with a suspensor, reaction to determine the localization of the total content of phenolic compounds (with Fast Blue reagent); (**B**)—reaction to flavans in the basal part of the cotyledons; (**C**)—reaction to flavans in the suspensor area, (**D**,**E**)—groups of embryo receptacle cells (idioblasts) with finely granulated and amorphous phenolic compounds (with Fast Blue reagent); (**F**)—groups of embryo receptacle cells (idioblasts) with flavans (reaction with vanillin reagent). **end**—endosperm; **em**—embryo; **sus**—suspensor; Scale bar: (**A**–**D**)—1 mm, (**E**,**F**)—0.01 mm.

Yew seeds are ovoid, and the surface is smooth with an oily sheen [38]. Depending on the stage of seed development, its color varies from green in young (Figure 3A,B,F,G) to dark brown in mature (Figure 4A,B and Figure 5A). The seed coat of yew is characterized by a well-developed mesoderm (sclerotesta) and a thick-walled epidermis with a very thick cuticle layer. Yew has an abundant endosperm, which makes up the bulk of the seed and is represented by large, tightly closed cells of the same size. The embryo in a mature seed is chlorophyll-free, straight, with two straight, underdeveloped cotyledons; a hypocotyl; and a root (Figure 6A). The size of the embryo is very small, about 3.5 × 0.3 mm. The embryo is located in the micropylar part of the endosperm along the central longitudinal axis of the seed. The apex of the shoot, enclosed between two cotyledons, is not differentiated and is represented by a group of meristem cells.

As yew seeds mature, the spatial localization of phenolic compounds practically does not change, but the intensity and severity of histochemical reactions change markedly. Even at the earliest stages of seed development, phenolic compounds are distributed throughout the cuticle, in the cells of the epidermis, hypodermis, and endoderm, mainly localized in the cell walls. A significant part of the polyphenols is concentrated in the area in the outer peripheral part of the nucellus and in the endosperm, in the area adjacent to the nucellus (Figure 3C,E,F,I). A vivid reaction to flavans is noted in the cuticle, hypodermis, outer mesoderm and endoderm, and is less evident in the inner mesoderm (Figure 3J–L). With the further development of the seed, by the end of the summer period, the reaction with the vanillin reagent to flavans becomes more intense, which is especially pronounced in the forming aril. Phenolic compounds, including flavans, were localized in scales, in the cell walls of the guard cells of stomata and conducting tissues (Figure 3M).

By the end of summer, the aril surrounding the yew seed becomes “fleshy” and massive and acquires a red color of varying degrees of intensity (Figure 4A,B). Staining for the presence of phenolic compounds is zonal in nature (Figure 4C). Among the ubiquitous but poorly expressed distribution of polyphenols, including the flavan series, in the parenchyma, there are “hyperaccumulative” zones, where phenolic compounds are found in intercellular areas, vacuoles and idioblasts, in the form of amorphous matter and granular material of varying degrees of aggregation. These zones with polyphenols are located along the upper edge of the aril, completely filling the cells of the epidermis and the underlying layer of the parenchyma (Figure 4H,I,L,M); they are unorganized in the thickness of the aril and in the area where the aril attaches to the chalaza (Figure 4E–G). The cells of the lower part of the aril, elongated along the longitudinal axis of the seed, contain phenolic compounds, including flavan compounds, in the form of large conglomerates, inclusions of varying degrees of granularity prone to aggregation, and in the form of an amorphous substance.

The upper and lower scales of the reproductive shoot, on which the seed with aril is located, contain polyphenols over their entire surface (Figure 4N,O). A pronounced accumulation of polyphenols was also noted in the area of attachment of scales to the reproductive shoot with seeds. There was no specific histochemical reaction mainly along the edges of the scales, but single cells with polyphenols, including flavans, were found in vacuoles and intercellular cells.

A study of the localization of polyphenols in yew seeds harvested in autumn showed that phenolic compounds represented by flavans and lignin are present in the cuticle and epidermis (Figure 5). Mesoderm sclereids also contain polyphenols everywhere (Figure 5B–I). The main part of polyphenols is found in preserved nucellus cells, where they are mainly found in the cell walls (Figure 5D,E,J–L). Previously, a qualitative study of the phenolic compounds of the seed revealed the presence of phenolic carboxylic acids, flavans and flavonols. Flavonoids in plant cells are mainly in a glycosidated form, that is, a peculiar form of sugar storage, and play a leading role in energy reactions associated with electron transfer [39,40]. Previously, researchers have shown differences in the taxol content in *Taxus* seeds. The highest content was observed in the mesoderm, followed by the endosperm and the embryo, respectively [41]. The taxol content reached its maximum at the middle stage of seed maturation and decreased with further maturation. The data presented on the taxol content in the seed during the growing season correlate with our data on the localization and quantitative content of polyphenols in the seed of plants of the genus *Taxus* [42].

Groups of host cells (idioblasts) with polyphenols were found in the basal part of yew cotyledons. Soluble phenolic substances are localized in them in the form of finely granular inclusions, and flavans are in the form of an amorphous substance (Figure 6A,D–F). Groups of phenol-accumulating cells with flavans are located not only in the basal part of the embryo (Figure 6B) but also in its suspension. In the micropylar part of the mature seed, a focal accumulation of phenol-accumulating cells with flavans in the form of an amorphous substance and granular inclusions was noted (Figure 6C). It should be noted that staining for the total content of phenolic compounds, in most cases, coincided with staining for flavans, which was previously confirmed by biochemical studies showing the dominance of the latter in the phenolic complex of Canadian yew seeds. This is probably due to the fact that seed metabolism is mainly directed towards the biosynthesis of reserve and physiologically active substances necessary for the long-term preservation of embryo viability during deep dormancy, which lasts from 1.5 to 3 years in yew seeds.

After conducting histochemical screening of the localization of polyphenols throughout the growing season, we can conclude the following:

1. The most intensively studied metabolites accumulate during the period of active growth and physiologically important events, such as preparation for fertilization.
2. Both female and male generative organs of yew contain large amounts of flavans (catechins and proanthocyanidins).
3. Seed formation and development are accompanied by a vivid reaction to polyphenols, especially in the integumentary tissues. The mature seed contains fewer areas with polyphenols, but they are clearly marked in the integumentary tissues and the area next to the embryo.
4. The embryo itself also contains zones accumulating polyphenols.
5. In the arillus, flavans are a dominant polyphenol class.

### 2.2. Cytotoxicity of Ethanolic Extracts from Various Parts of Plant Taxus

Our investigation into the cytotoxicity of ethanolic extracts from various *Taxus* species determined the IC50 values (the concentration inhibiting 50% of cell viability). The data presented in Table 1 clearly demonstrate that the ethanolic extracts from all studied yew species exhibited low toxicity towards normal FetMSC cells, which is undoubtedly a crucial factor for potential therapeutic applications. HepG2 liver carcinoma cells also showed considerable resistance to the action of the yew ethanolic extracts.

However, the extracts from the shoots of *T. canadensis* displayed slight, nearly identical cytotoxicity against this cell line. In all tested scenarios, the most sensitive cell lines to the ethanolic yew extracts were HeLa (human cervical adenocarcinoma, clone M) and A-172 (human glioblastoma).

It was demonstrated that the extractive substances obtained from the shoots of *T. canadensis* possessed the greatest cytotoxic effect among the samples tested.

**Figure 7:**
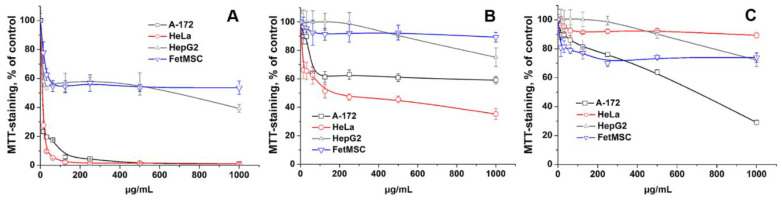
Comparison of the cytotoxicity of ethanolic extracts of shoots (**A**), mature seeds (**B**) and aril (**C**) of Canadian yew on various lines of tumor (HepG2, HeLa and A-172) and normal (FetMSC—mesenchymal stem cells from embryonic bone marrow) cells.

A diagram (Figure 7) shows the effect of yew ethanolic extract at a concentration of 15 μg/mL on the survival of normal and tumor cells. The comparison of different extracts reveals that ethanolic extract No. 1, derived from *T. canadensis* shoots, had the most significant cytotoxic effect on tumor cells compared to other yew ethanolic extracts.

The yew seed is surrounded by a fleshy, flanked roof (aril), which changes color from green to bright red as the seed matures (Figure 4). Based on the literature data, it is the aril, rich in secondary metabolites, that determines the conditional edibility of mature yew “berries” for some bird species, which may be indirect evidence that the aril has the lowest content/absence of taxol compared to the rest of the plant. Using the methods of chemotoxic analysis, it will be possible to speak more confidently about the safety of the aril of the genus *Taxus*.

### 2.3. Antifungal Activity of Taxus Plant Ethanolic Extracts

**Figure 8:**
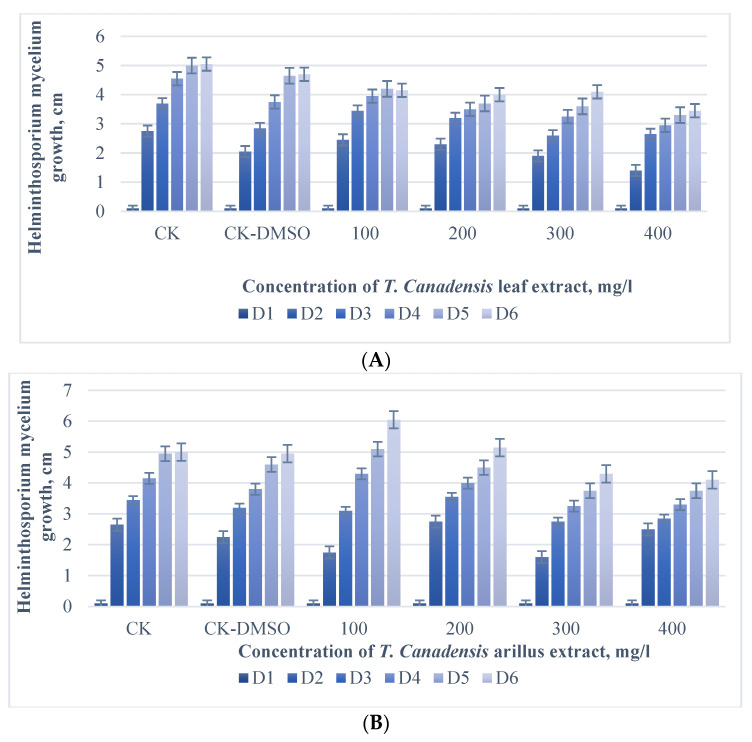
The effect of ethanolic extracts of *T. canadensis* leaf (**A**) and aril (**B**) on the growth characteristics of *Helminthosporium sativum* L. mycelium.

When working with ethanolic extracts of Canadian yew (from leaves and aril), the inhibition of the growth of *Helminthosporium sativum* L. was noted in all experimental groups compared to the control group. With an increase in the concentration of the ethanolic extract, the inhibition of the growth of *Helminthosporium sativum* L. increased and reached its maximum value at a concentration of 400 mg/L (Figure 8).

**Figure 9:**
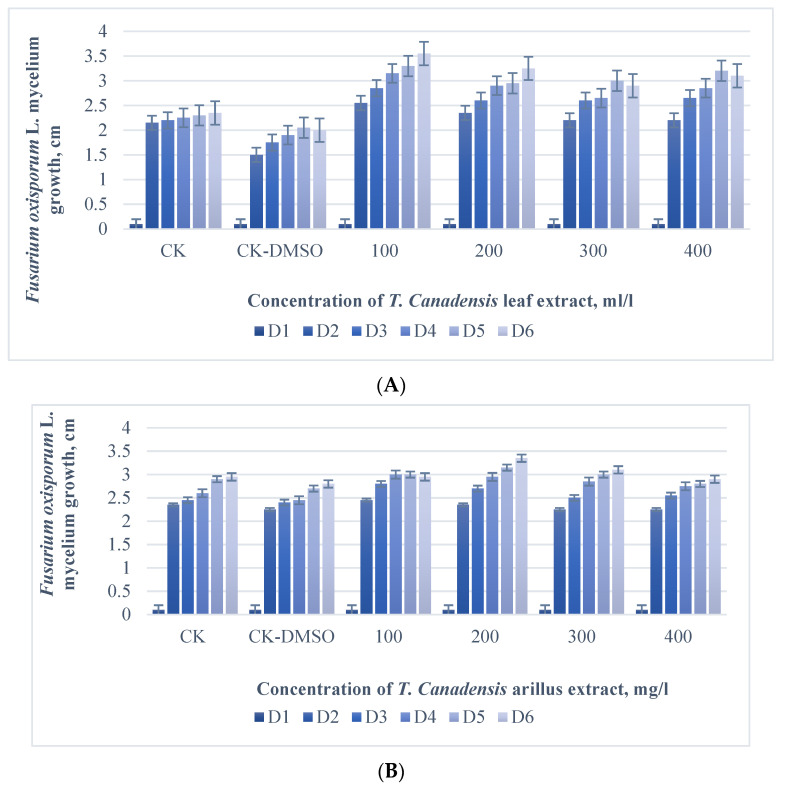
The effect of ethanolic extracts of *T. canadensis* leaf (**A**) and aril (**B**) on the growth characteristics of *Fusarium oxisporum* L. mycelium.

The most significant results were noted for the ethanolic extracts from yew leaves. Compared with the control group, the phenomenon of stimulating the growth of *Fusarium oxisporum* L. was observed in each experimental group (Figure 9) under the action of extractive substances.

### 2.4. Antibacterial Activity of Taxus Plant Ethanolic Extracts

**Figure 10:**
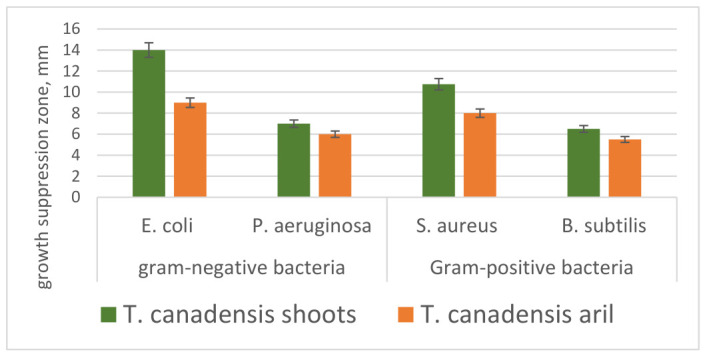
Antibacterial activity of *Taxus canadensis* L. shoots and aril ethanolic extract.

Extractive substances of *T. canadensis* show high antibacterial activity, especially against *E. coli* and *S. aureus* (Figure 10). The average diameter of the inhibition zone for *E. coli* was 14.03 ± 0.17 mm. Scientists have previously shown that the active components of plant secondary metabolism can destroy the microbial cytoskeleton and disrupt energy metabolism [43]. We can observe this by the different physiological responses from different representatives of bacteria to the identical composition of ethanolic extracts. Our next studies will involve an in-depth study of key antibacterial components and their molecular mechanisms of action.

### 2.5. The Content of Polyphenols in Ethanolic Extracts of Taxus canadensis L.

**Figure 11:**
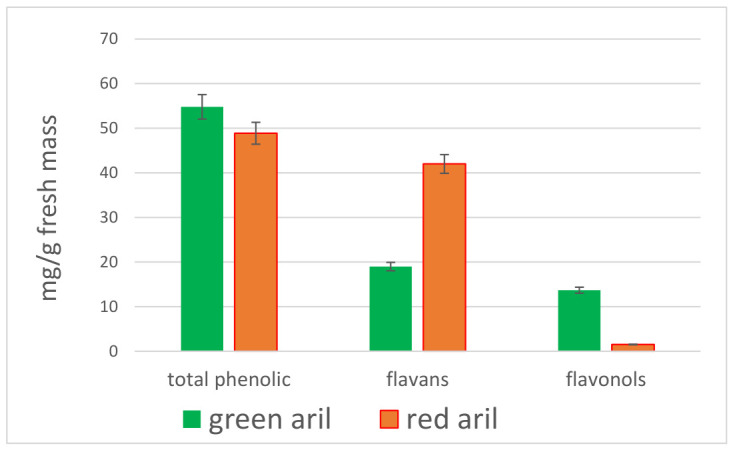
Accumulation of polyphenols in the shoots and aril of *Taxus canadensis* L. According to all three indicators, the differences between the “green aril” and “red aril” groups are statistically significant (*p* < 0.05).

During the growing season, as the aril matures, the content of polyphenols changes (Figure 11). Against the background of a slight decrease in the total content of phenolic compounds in mature red aril, the proportion of flavans increases. When the aril matures, the proportion of flavanols decreases—the most common polyphenols, the biosynthesis of which is confined to chloroplasts. Substances of flavan nature were predominantly in the aril at various stages of vegetation, which is confirmed by our histochemical studies.

The use of standard taps, as well as qualitative reactions to various groups of phenolic compounds, made it possible to preliminarily identify some of the substances present in ethanolic extracts of yew seeds. These include substances of a flavan nature, represented by simple forms of catechins, such as (+)-catechin and (−)-epicatechin, as well as proanthocyanidins (oligomeric derivatives of (+)-catechin and (−)-epicatechin). Among the flavonols were rutin, naringenin, as well as kaempferol and quercetin glycosides. Phenylpropanoids are represented by p-coumaric, oxybenzoic, gentisic, vanillic, ferulic, chlorogenic and caffeic acids, as well as other derivatives that require further identification.

Plants of the genus *Taxus* are relict slow-growing species that have been living for more than 3000 years with a limited and inaccessible distribution range in various ecological, geographical and floristic areas. The natural reproduction of yew is difficult, since its seeds are characterized by a combined dormancy (deep physiological rest, underdevelopment of the embryo and hard-seeding), which requires complex pre-sowing preparation for germination. In central Russia, these species grow only in botanical gardens and private landscaping. The Canadian yew is a shrub up to 2 m tall that grows in the undergrowth of dark coniferous forests of hemlock, spruce and thuja in North America. The color of its needles is yellow-green, which turns into a terracotta shade in winter.

The Canadian yew is characterized by the highest winter hardiness and shade tolerance of all yew species. Determining the localization of polyphenols is an integral part of studying the phenolic metabolism of plants. Although the formation of polyphenols is inherent in all organs and tissues of plants, they can have different compositions, concentrations and localization due to physiological functions. Previously, we have shown a significant number of polyphenols in yew shoots and seeds. The phenolic complex of its generative organs is represented by both phenylpropanoids and flavonoids [42,44]. In addition, Canadian yew flavans are the dominant components of the phenolic complex, accounting for up to 70% of the amount of soluble phenolic compounds. Most likely, it is the presence of flavans that determines the high winter resilience and unpretentiousness of Canadian yew compared to other representatives of this genus. Flavonoids are the most highly reactive low molecular weight substances with strong antioxidant and cryoprotective properties.

In recent years, when the resistance of pathogens to antibiotics and antimicrobials is becoming more serious, the search for new and natural remedies has become particularly urgent [45]. Thus, the study of the fungicidal and antibacterial activity of different plant species, for example *Taxus* plant in new regions (Moscow), may provide a scientific basis for developing new natural inhibitory growth pathogens. This will help us to understand the biological features and potential value of the application of different parts of these unique plants [46]. As seen, even within a single species of yew, the chemical composition and biological activity of different plant parts can vary greatly. Chemotaxonomy allows plants to be classified and identified according to proven differences and similarities in their biochemical composition. In our case, it is demonstrated that different *Taxus* organs exhibit different characteristics when suppressing cancer cell growth. Even within the generative organ, the cytotoxicity of semen and aril extracts is differentiated. The influence of extractive substances on the mycelial growth of pathogenic fungi is heterogeneous. Selection of plants based on chemotaxonomics is a prerequisite for successful research into the potential use of plants to obtain target substances [47]. By comparing the antimycotic spectrum of extracts from relict woody plants of different species, we hope to identify chemotaxonomic diversity. The specificity of the antimicrobial activity of extracts of *Taxus* may provide a scientific basis for further development of biopreparations based on it.

As is known, the secondary metabolites of plants have an influence not only on the growth processes of the plant organism in general, but also exhibit allelopathic and fungicidal properties [48]. It can be assumed that different ethanolic extracts of yew, obtained at different stages of vegetation, have a stronger inhibitory effect on certain bacteria. It has been shown that “extractive substances of the core” of coniferous trees have fungicidal, bactericidal or insecticidal properties [49]. Ethanolic extracts rich in numerous organic compounds determine the durability and specific coloring of “red tree” wood. Our studies also show fungicide activity; these extracts are rich in secondary metabolites. The histochemical studies we have carried out on the reproductive organs show for the first time an inward distribution of substances with high biological activity—polyphenols.

The study shows that ethanolic extracts of *Taxus* from generative and vegetative tissues have some complex biological potential. However, the effectiveness of ethanolic extracts is influenced by many metabolic factors with respect to polyphenols accumulation [50,51]. Our future research will further consider the influence of these factors on the fungicidal and bactericidal effects of ethanolic extracts. All of this will help to optimize the natural resources of this for developing new antimicrobial agents and cytostatics.

The pleiotropic anticancer effectiveness of phytochemical natural substances (polyphenols) is manifested in slowing down or reversing the process of metastasis, as well as in preventing invasiveness and metastasis. The effects of flavonoids against cancer are associated not only with early stages of the cancer process but also with cancer progression and spread into distant sites [31].

The researchers note the need to introduce flavonoids into clinical research with a focus on a targeted and personalized approach to fighting cancer. Depending on their chemical structure, degree of oxidation, and nature of substitution of the heterocyclic pyran ring, flavonoids can have a wide therapeutic effect. For example, the flavone apigenin has an antimetastatic effect by inhibiting the nuclear organization and transcriptional activity of the STAT3 protein [31,52]. Quercetin also inhibited STAT3 transcription activity and target genes that are involved in cell growth, migration, and invasion in melanoma A375 and A2058 cells [53]. Luteolin decreased the pleiotrophin (PTN) expression, a gene positively related to cancer progression [54]. It should not be forgotten that the bioavailability and biological activity of flavonoids are strongly influenced by metabolic processes [55].

## 3. Materials and Methods

The object of the research was the generative organs of the Canadian yew (*Taxus canadensis* Marsh.), collected during the entire growing season (April–October) from plants growing in the Moscow region.

For histochemical analysis, live fresh plant material of the Canadian yew was used as research objects: microstrobiles, megastrobiles, microsporophylls, ovules and seeds with arils at different stages of maturation, as well as isolated embryos. The localization of phenolic compounds on temporary preparations was determined by histochemical methods: for the amount of phenolic compounds, the material was stained with a 0.08% solution of Fast Blue reagent. To study the localization of flavans (catechins and proanthocyanidins), a reaction with a vanillin reagent in hydrochloric acid vapors was used. In order to preserve the intracellular distribution of phenolic compounds, all reactions were performed in nonpolar solvents [56]. The preparations were examined using a light microscope (“Karl Ziess”).

To extract phenolic compounds, the plant material (1000 mg) was crushed and then subjected to extraction with hot 96% ethanol (1 mL). The extracts were spectrophotometrically determined for the sum of soluble phenolic compounds (with Folin-Denis reagent), flavans (with vanillin reagent), and flavonols (with aluminum chloride). Calibration curves for determining the total content of soluble phenolic compounds and flavans were constructed using (−)-epicatechin, and for determining flavonols, they were constructed using routine [45].

For a preliminary study of the composition of phenolic compounds, ethanolic extracts of plant material were evaporated dry (humidity 10–12%). The residue was dissolved in a small volume of ethanol and the resulting solution was subjected to one–dimensional chromatography in a thin layer (0.25 mm) of microcrystalline cellulose (solvent: n-butanol + CH_3_COOH + water, 40:12:28). The identification of substances was carried out on the basis of specific fluorescence data in UV light (wavelength 254 and 365 nm), by Rf values compared with standard taps, as well as using qualitative reactions with a number of specific reagents: a mixture of ferric chloride and potassium ferric chloride (for all classes of phenolic compounds), vanillin reagent (for flavans), aluminum chloride (for flavonoids) and diazotirovannym para-nitroaniline (for phenolic carboxylic acids) [57].

### 3.1. Preparation of Ethanolic Extract for Determination of Biological Activity

The extracts were prepared from the raw biomass of shoots, seeds and leaves of Canadian yews, where the weight of the sample was 1 g. In total, 10 mL of 96% ethyl alcohol was added to the crushed vegetable raw materials and extracted in the dark by ultrasound (35 kHz) for an hour at a temperature of 37.1 °C. The resulting extract was centrifuged for 3 min and filtered through a paper filter (blue stripe). After filtration, the extracts were used for bacteriostatic and antifungal studies.

To determine the cytotoxic properties, the extract obtained by the above method was dried using a vacuum evaporator and weighed to determine the exact mass. The dry residue (humidity 10–12%) was used to work with cancer cells.

### 3.2. Cell Cultures and Cytotoxicity Assay

The cytotoxic properties of extracts were studied using an MTF assay. Cells were seeded into 96-well culture plates in concentrations of 5104 cells/mL for HeLa and A-172, 7104 cells/mL for HepG2, 105 cells/mL for FetMSC cells. The dry residue ethanolic extracts were dissolved in 96% ethyl alcohol to a final concentration of 100 mg/mL dry matter. To 990 mL of cultural medium was added 10 mL of alcohol solution up to a final concentration of 1000 μg/mL. The extracts were added to the nutrient medium after 24 h of cultivation. Cytotoxicity of extracts was assessed at exposure for 72 h in the concentration range 1–1000 ug/mL. At the end of the experiment, cells were incubated for 3 h in the presence of the dye 3-(4,5-dimethylthiaziol-2-yl)-2,5-biphenyl-2N-tetrazolium bromide (MTT, DeAM, Russia) at a concentration of 0.5 mg/mL. The resulting formalae crystals were dissolved in 100% DMSO. Optical density was measured at 570 nm and 620 nm wavelengths using the multifunctional Spark 10M flatbed reader (Tecan, Switzerland). The cytotoxicity index (IC50) was determined by a dose–response curve using median effect analysis.

### 3.3. Antifungal Activity Assay

For the work on primary screening of Antifungal activity, desiccated ethanolic extracts (humidity 10–12%) obtained from shoots and aril equal to 1 g were used. All experiments were carried out on pure cultures of fungi of the genus *Fusarium oxisporum* L. and *Helminthosporium sativum* L.

These strains were identified and separated by the staff of the Laboratory of Mycology of the Institute of Phytopathology of the Russian Academy of Sciences. For the experiment, live fungus cultures, long stored in a refrigerator at a temperature of +4 °C, were used, which were initially multiplied in a nutritious environment containing mineral salts on a phyto-hormone-free basis. The fungi were grown in Petri dishes on a solid nutrient medium (1/2 MS) according to the previously described method in a light room at a temperature of 25 °C, with a photoperiod of 16 h and an intensity of 3000 lux [43].

Dry plant extractive residue, obtained from evaporated ethanolic extracts. For this 1-g panel of plant material, 10 mL 96% ethyl alcohol was extracted over an hour at a temperature of 37 °C, filtered through a paper filter (blue stripe), dried and weighed to determine the exact mass. The lyophilized dry extractive residue hanger was dissolved in DMSO and added to the composition of the nourishing medium after autoclaving. The extract concentration was 100, 200, 300, 400 mg/L. Control served an environment without the extract as well as a pure solvent (DMSO). For control in the study of fungicide activity, distilled water and dimethyl sulfoxide (DMSO)—a strong chemical solvent—were used. DMSO is rapidly absorbed by transdermal administration and exhibits low toxicity. This property can be used as a means of transdermally delivering poorly dissolved drugs in other solvents directly to their site of action. In addition, this antioxidant is known as a growth inhibitor of pathogens with antifungal and antimicrobial action, cryopreservative, analgesic and anti-inflammatory. As a consequence, its effect on the mycopathogen was studied separately compared to the control of water.

Fungicidal activity of ethanolic plant extracts was determined by mycelial growth of the fungus, measuring the diameter of the fungus in two planes. Plates were incubated at 25 °C for 7 days, and the inhibition zones were measured [57]. During the cultivation period, the diameter of the fungus was measured in two planes.

### 3.4. Antibacterial Activity Assay

The antibacterial activity was determined by the disk method. Investigated strains of bacteria (*Escherichia coli*, *Pseudomonas aeruginosa*, *Bacillus subtilis*, and *Staphylococcus aureus*) were obtained from the Department of Microbiology, Moscow Timiryazev Agricultural Academy. Bacterial isolates were transplanted onto a dense agar medium and cultured at 37 °C for 24 h. Colonies were selected with a bacteriological loop and placed in sterile distilled water, compared with the turbidity standard (0.5 McFarland standard). After agitation, 200 µL of the prepared bacterial suspension was taken and placed on a plate of nutrient agar in a Petri dish, evenly distributed with a sterile spatula. Two-layer sterile discs made of filter paper with a diameter of 6 mm were impregnated with ethanolic extracts and laid out on the surface of the agar with the applied bacterial culture. In total, 40 µL of freshly prepared ethanolic extracts of plant leaves (1 g of fresh plant material extracted with 10 mL of hot 96% ethyl alcohol over an hour in the dark) was used to impregnate the discs. Then, 96% ethanol was used as a negative control. After cultivation at 37 °C for 24 h, the diameter of the inhibition zone was measured. The experiment was carried out in three repetitions.

### 3.5. Statistical Analysis

Data are presented as mean ± standard deviation. IC_50_ values were calculated using median-effect analysis. All studies were conducted in three biological and five analytical replicates.

## 4. Conclusions

It has been shown that the examined organs of the Canadian yew have a high capacity for biosynthesis of various secondary compounds. Phenolic compounds form both simple structures and their polymeric forms. Judging by the histochemical reaction, flavonoids are the dominant components of the phenolic complex. Megastrobiles, microstrobiles, nucleus, integument, micropile, and megaphone contain phenolic compounds in idioblasts in vacuoles, cell walls, and intercellular spaces. In seeds, the main part of polyphenols is localized in the exothesis, predominantly in epidermal layers, in the endosperm adjacent to the embryo (in cell walls), in idioblasts of the basal part of the cotyledons, and in the peduncle of the embryo. Plants of the genus *Taxus* are characterized by the formation of a mature seed, fleshy pulp—the arillus. Yew cones are rich in metabolites of primary and secondary origin. The work showed that during the whole period of vegetation and at various stages of development in the aril, secondary compounds are actively formed, which were localized in the covered tissues, runners, and also some of the resinous and slime receptacles of the aril. Flavans are the dominant components of the phenolic complex aril.

Ethanolic extracts from the vegetative plant organs of *Taxus* have a pronounced cytotoxic activity. While the extractive substances from the aril showed no cytotoxic effects, indicating its low toxicity and conditional edibility. It is likely that the seed causes the toxicity of *Taxus* fructification.

It is known that secondary metabolites act as mediators in the relationship between plants, the environment and microorganisms. The study found that ethanolic extracts from summer vegetative period offshoots and from the ripe seeds (red aril) of the Canadian yew had pronounced fungicidal activity. The growth of mycelium in pathogenic fungi depends on the concentration of the ethanolic extract and the strain of the phytopathogen under investigation. The ethanolic extracts investigated had a toxic effect on the growth of the mycelial fungi *Helminthosporium*. The plant ethanolic extract had a maximum inhibitory effect on *Helminthosporium* at a concentration of 300–400 mg/L. However, for *Fusarium oxisporum* L., we observe a slight stimulating effect of the ethanolic extracts of this on mycelial development. The excretory substances of the plants of *Taxus* inhibit the growth of bacteria, and the greatest bacteriostatic effect is noted for *E. coli*.

Based on the data presented, it can be concluded that the extractive substances of both the vegetative and generative organs of *Taxus* can find their application in various industries, including pharmacokinetics. The specificity of the antimicrobial activity of extracts of *Taxus* may provide a scientific basis for further development of biopreparations based on it. Current research highlights the need for novel delivery systems, such as nanoparticles and liposomes, to enhance systemic absorption and therapeutic efficacy. Polyphenols hold promise as natural anticancer agents, with the potential to complement conventional therapies and improve patient outcomes. This work may be continued in the development of ways to increase the stability and bioavailability of species-specific metabolites. The subsequent manufacture of nanocapsules, such as liposomes with yew polyphenols, and obtaining data to study their interaction with enzymes during digestion in vivo may open up prospects for further use in phytotherapy and the food industry [58]. Despite promising research results, the biological and therapeutic potential of flavonoids, and especially procyanidins, has not been sufficiently studied. A clear definition of the localization of various groups of secondary metabolites and the vegetative timing of their formation in poorly studied generative organs of yew plants provides important information for phytotherapists.

## Figures and Tables

**Table 1:** Comparison of cytotoxic effects of ethanolic extracts obtained from *T. canadensis* plants. IC_50_ values of yew ethanolic extracts for various human cells after 72 h of exposure.

Sample	Concentration Inhibiting Cell Viability by 50% IC_50_, μg/mL			
	FetMSC	HepG2	HeLa	A-172
Shoot	>1000	636.0 ± 31.6	5.7 ± 0.4	6.2 ± 0.3
Aril	>1000	>1000	>1000	602.1 ± 27.1
Ripe seed	>1000	>1000	189.7 ± 11.6	>1000

## Data Availability

The original contributions presented in this study are included in the article. Further inquiries can be directed to the corresponding author.
